# Transcription factor CsWRKY40 regulates L-theanine hydrolysis by activating the *CsPDX2.1* promoter in tea leaves during withering

**DOI:** 10.1093/hr/uhac025

**Published:** 2022-02-19

**Authors:** Haiyan Cheng, Wei Wu, Xiaofen Liu, Yuefei Wang, Ping Xu

**Affiliations:** 1 Institute of Tea Science, Zhejiang University, Hangzhou 310058, Zhejiang, China; 2 Key Laboratory of Horticultural Plant Growth, Development and Quality Improvement, Ministry of Agriculture, Hangzhou 310058, Zhejiang, China; 3 Zhejiang Provincial Key Laboratory of Horticultural Plant Integrative Biology, Zhejiang University, Hangzhou 310058, Zhejiang, China

## Abstract

L-Theanine is a crucial secondary metabolite in tea and positively determines the potential quality and health benefits of tea products. Previous work found that the content of L-theanine decreased during the withering process, although the specific mechanism is still unknown. Here, weighted gene co-expression network analysis (WGCNA) was performed based on transcriptome data obtained previously. The key hydrolysis gene *CsPDX2.1* in L-theanine metabolism and seven candidate transcription factors were screened out. Among these transcription factors, CsWRKY40 displayed the strongest activation on the *CsPDX2.1* promoter (373.18-fold) by binding to the W box element, based on dual luciferase assay and EMSA results. The CsWRKY40 protein was located in the nucleoplasm, whereas CsPDX2.1 was found in both the nucleoplasm and cytoplasm. Analysis of withering, water-retention, and water-loss treatments confirmed that water loss from tea leaves was the critical factor that affected ABA and L-theanine contents by activating the expression of *CsWRKY40* and *CsPDX2.1*. Our results provide a new insight into the regulatory mechanism of L-theanine hydrolysis metabolism.

## Introduction

Secondary metabolites, ubiquitous molecular substances involved in plant growth and stress responses, are also crucial to the quality and physiological functions of plant products [1–3]. The abundant secondary metabolites in tea (*Camellia sinensis* (L*.*) O. Kuntze) give its products a unique flavor and remarkable biological activity, making it one of the most widely consumed beverages around the world [4, 5]. As one of the specialized secondary metabolites in tea, L-theanine (γ-glutamylethylamine) accounts for 40–70% of the free amino acids in tea leaves [6]. The L-theanine content directly influences the flavor of tea infusions, especially for green tea, because of its umami taste [7, 8]. L-theanine also contributes to the formation of key volatiles during tea processing [9, 10]. It has been found to have various health benefits, including neuroprotection, relaxation, improvement of memory and learning performance, and prevention of cancer and the common cold [11–13].

Since the 1960s, many studies have been carried out on the metabolism of L-theanine in tea plants [14, 15]. L-theanine is synthesized from glutamic acid and ethylamine, mainly in the tea plant roots, subsequently transported to the young shoots through amino acid permease family members (AAPs), and finally hydrolyzed in leaves [6, 15]. Recently, Cheng et al. [16] found that higher L-theanine accumulation in albino tea leaves was due to weak catabolism of L-theanine rather than activation of L-theanine biosynthesis. Furthermore, the hydrolysis of L-theanine occurs not only in tea plants but also in tea leaves plucked for processing. Withering is the first step of tea processing for green, black, oolong, and other teas, during which the harvested tea leaves are spread and aired evenly in a specific environment for several hours [17]. Previous studies, including our own research, have shown that the content of L-theanine decreases during withering [18, 19]. Moreover, our recent work indicated that L-theanine metabolism could be regulated by endogenous ABA [20].

**Figure 1:**
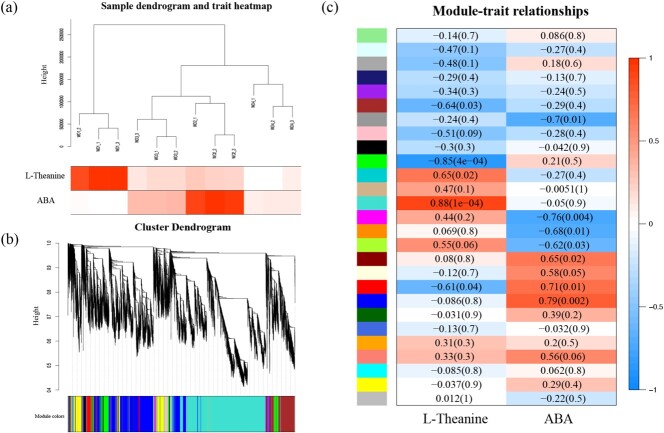
Cluster analysis of samples and construction of the co-expression modules by WGCNA. (a) Sample dendrogram and trait heatmap based on the gene expression and physiological data. (b) Gene cluster dendrogram based on the topological overlap with 27 different modules. (c) Correlation analysis of the modules and traits. *P*-values are shown in parentheses.

However, less is known about L-theanine hydrolysis than about its biosynthesis, as the related genes remain largely unknown [21]. Recently, *CsPDX2.1* was reported to be the L-theanine hydrolysis gene based on its hydrolysis ability *in vitro* [22], providing an important basis for revealing the transcriptional regulatory mechanism of L-theanine hydrolysis. Nevertheless, the critical transcription factors involved in regulating L-theanine hydrolysis and its specific mechanism have yet to be determined. Here, using previously obtained data, we performed weighted gene co-expression network analysis (WGCNA) and confirmed that the key gene in L-theanine metabolism during withering was *CsPDX2.1*. Dual-luciferase assay and electrophoretic mobility shift assay (EMSA) results indicated that the transcription factor (TF) CsWRKY40 could directly bind to and activate the promoter of *CsPDX2.1*. Withering, water-retention, and water-loss treatments were applied to tea leaves, and they confirmed that water loss from tea leaves was the key factor leading to L-theanine hydrolysis.

## Results

### WGCNA and isolation of candidate TFs

After filtering out genes with low expression, 26 524 genes (FPKM ≥ 20 in all samples) were retained. Co-expression networks were constructed with L-theanine and ABA, respectively. The sample dendrogram and trait heatmap were visualized to evaluate the relationship between the relevant gene expression data and the physiological traits within and between groups (Fig. 1a). The expression patterns of all genes were distinguished and categorized to generate a cluster dendrogram with 27 modules (Fig. 1b). The diagram of module-trait relationships indicated that the turquoise module and the blue module showed significant correlations with L-theanine and ABA, with Pearson correlation coefficients of 0.88 and 0.79 (P < 0.01), respectively (Fig. 1c).

Based on gene annotation data, two genes, *MSTRG.33795* and *MSTRG.9423*, associated with L-theanine metabolism were obtained from the blue module (6726 genes). According to the RNA-seq data, the FPKM value of *MSTRG.33795* was much higher than that of *MSTRG.9423* (Fig. S1). NCBI BLAST analysis of the full amino acid sequence revealed that *MSTRG.33795* was *CsPDX2.1*. No genes related to L-theanine metabolism were found in the turquoise module, suggesting that the gene expression pattern differed from the pattern of change in L-theanine content. Through further screening, 344 genes were found to be highly correlated with *CsPDX2.1.* Among them, seven unigenes encoded putative TFs related to *CsPDX2.1* (Fig. S2). Finally, six genes were obtained after full-length cloning and sequence analysis: *CsWRKY40* (CSS004414), *CsbZIP* (CSS005984), *CsB3* (CSS020472), *CsbHLH* (CSS003725), *CsGRAS* (CSS030401), and *CsZF* (CSS013198).

### *In vivo* regulatory effects of selected TFs on the *CsPDX2.1* promoter

**Figure 2:**
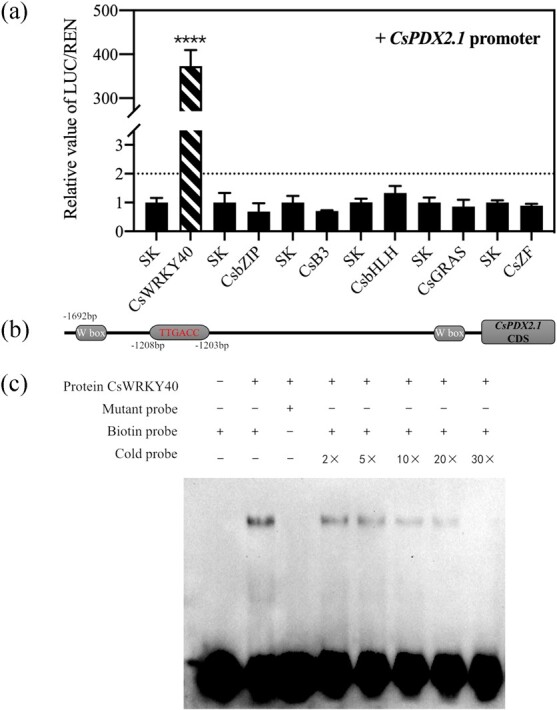
Regulatory effects of six TFs on the *CsPDX2.1* promoter and EMSA analysis of the binding ability of CsWRKY40 to the *CsPDX2.1* promoter. (a) The Luc/Ren ratios of various TFs are all relative to the value of empty vector plus promoter, which was set to 1. Values are means (+SE) from three biological replicates (^****^P < 0.0001). (b) The locations of the three W boxes in the promoter are shown, and the protein binding sequences and sites are highlighted. (c) Purified protein was mixed with three probes for EMSA. The presence (+) or absence (−) is shown. The biotin probe and mutant probe contained a 3′ biotin label, and the cold probe lacked the 3′ biotin label. The binding ability of the protein was analyzed by the concentration ratio of the cold probe to the biotin probe.

Dual-luciferase assays showed that there was extremely significant transactivation (373.18-fold) by CsWRKY40 on the *CsPDX2.1* promoter, whereas other TFs had little effect (Fig. 2a). The *CsPDX2.1* promoter sequence contains three W boxes, which are located at −145 bp to −140 bp, −1208 bp to −1203 bp, and −1607 bp to −1602 bp. EMSA results showed that the *CsWRKY40* protein bound to the second W box (TTGACC) (Fig. 2b). The purified *CsWRKY40* protein delayed the biotinylated probe, and the binding affinity was gradually reduced by an increasing concentration of cold probe (without biotin labeling). Additionally, the presence of *CsWRKY40* protein had no significant effect on the mobility of the mutant biotinylated probe (Fig. 2c).

### Bioinformatics analysis of *CsWRKY40*

Sequence analysis indicated that *CsWRKY40* belonged to Group IIa of the WRKY family. To investigate the relationships of CsWRKY40 to Group II WRKY proteins from *Arabidopsis*, a phylogenetic tree was constructed using MEGA X (Fig. S3a). The results showed three Group IIa WRKY proteins, AtWRKY60, AtWRKY18, and AtWRKY40, in the *Arabidopsis* database. Among them, AtWRKY40 had the highest homology to the CsWRKY40 protein. Multiple sequence alignment showed that CsWRKY40 contained two highly conserved WRKY DNA-binding domains, the WRKYGQK motif and a CX_1-5_CX_22-23_HXH zinc finger, consistent with those of five other Group IIa WRKY proteins from *Arabidopsis*, *kiwifruit,* and *Cinnamomum camphora* (Fig. S3b).

### Subcellular localization analysis of CsWRKY40 and CsPDX2.1

**Figure 3:**
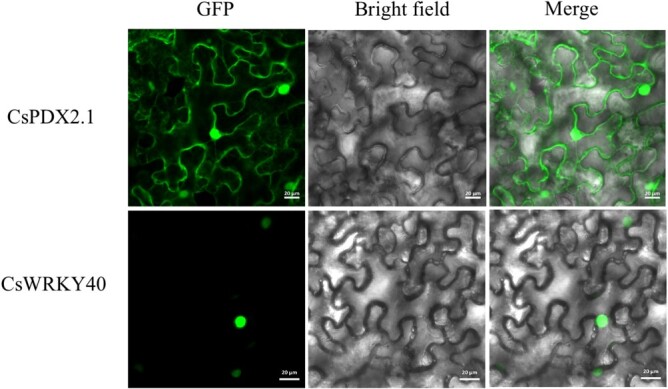
Subcellular localization of CsPDX2.1-GFP and CsWRKY40-GFP in *N. benthamiana* leaves. Green fluorescence represents the localization of the fusion protein. The GFP fusion protein was found in the cytoplasm and nucleoplasm structure of tobacco leaf cells, whereas the CsWRKY40-GFP fusion protein was found in the nucleoplasm structure. Bars = 20 μm.

To determine the subcellular localization of CsWRKY40 and CsPDX2.1, transient expression assays in *N. benthamiana* leaves were used to visualize the locations of the target proteins. The green fluorescence signals of CsWRKY40 were clearly distributed in the nucleus, whereas those of CsPDX2.1 were located in both the cytoplasm and nucleoplasm (Fig. 3), consistent with a previous result [22].

### Moisture, L-theanine, and endogenous ABA contents in three treatments

**Figure 4:**
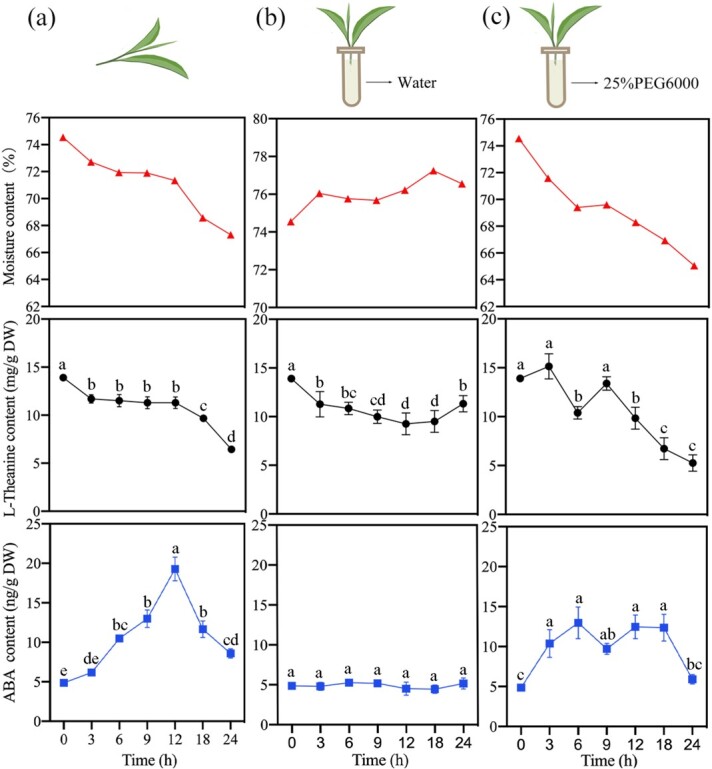
Changes in water, L-theanine, and ABA contents in three treatments within 24 h. The tender tea stems were placed on plates to wither naturally (a), inserted into tubes filled with water for the water retention (b), or inserted into tubes filled with 25% PEG6000 for the water loss (c). Different letters indicate significant differences among treatments (P < 0.05).

Previously, we found that L-theanine decreased gradually with the loss of water during withering, and transcriptome analysis showed that ABA might potentially regulate L-theanine metabolism [20]. Therefore, to further investigate whether moisture content was the key factor involved in L-theanine catabolism in tea leaves, we established three treatments, a withering treatment, a water retention treatment, and a water loss treatment. The moisture, L-theanine, and ABA contents of tea leaves were measured at 0, 3, 6, 9, 12, 18, and 24 h. In the withering treatment (Fig. 4a), the contents of moisture (from 74.544% to 67.313%) and L-theanine (from 13.903 mg g^−1^ to 6.442 mg g^−1^) decreased significantly in tea leaves with a similar trend over 24 h, whereas the ABA content first increased and then decreased, reaching a peak at 12 h (average of 19.285 ng g^−1^ DW). When water was continuously supplied to tea leaves, the levels of L-theanine and ABA were basically unchanged (Fig. 4b). The water loss treatment created a drought stress during which tea leaves stably and continuously lost water. The results showed that the water content (from 74.544% to 65.052%) and L-theanine content (from 13.903 mg g^−1^ to 5.260 mg g^−1^) continued to decline, and their variation ranges were greater than those in the withering treatment. Meanwhile, ABA content increased significantly from 0 h (average of 4.875 ng g^−1^ DW) to 6 h (average of 12.965 ng g^−1^ DW) and was then maintained until it decreased dramatically at 24 h (average of 5.911 ng g^−1^ DW) (Fig. 4c). The highest ABA content in the water loss treatment was not higher than that in the withering treatment; a possible reason was that the rapid loss of water in a short period of time led to an insufficient ABA response. These results indicated that water content in tea leaves was directly related to L-theanine metabolism and negatively regulated ABA metabolism to a certain extent.

**Figure 5:**
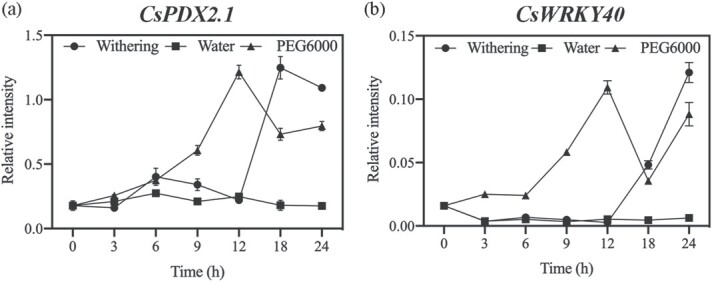
Expression of *CsPDX2.1* and *CsWRKY40* in three treatments. Values are means (±SE) from three biological replicates.

### Expression of *CsPDX2.1* and *CsWRKY40* in three treatments

The expression levels of *CsPDX2.1* (Fig. 5a) and *CsWRKY40* (Fig. 5b) were determined in samples from the three treatments. qRT-PCR analysis showed that *CsPDX2.1* was clearly positively correlated with *CsWRKY40*. In the case of sufficient water supply, the expression levels of both genes remained stable. While the leaves were under drought stress, gene expression levels increased significantly within 12 h and were higher than those in the withering treatment.

## Discussion

L-theanine is an important contributor to the quality and bioactivity of tea [23]. Therefore, understanding the mechanism of L-theanine hydrolysis during withering is crucial for maintaining L-theanine content during processing. Although previous studies indicated that *CsPDX2.1* was the L-theanine hydrolase gene [22], the key transcription factors and regulatory mechanisms that regulate *CsPDX2.1* have not yet been reported. Based on transcriptome analysis, we confirmed that *CsPDX2.1* was a key gene for L-theanine hydrolysis during withering, and further study identified CsWRKY40 and verified that it had a strong transcriptional activation effect on *CsPDX2.1*.

### *CsPDX2.1* is the key gene of L-theanine hydrolysis during withering, and six candidate TFs were obtained

WGCNA is thought to be an effective tool for identifying key genes and TFs of target metabolites from large amounts of transcriptome data. For instance, Wang et al. [24] used WGCNA to construct a network using 13 010 genes and 22 tea metabolites, ultimately identifying *CsMYB* as a key TF involved in lipid and flavonoid metabolism under blue light. Using the WGCNA method, we constructed co-expression networks by associating the transcriptome data with L-theanine content and ABA content during withering (Fig. 1). By screening the genes in the most correlated modules, no genes related to L-theanine synthesis were found in the two modules, but the L-theanine hydrolysis gene *CsPDX2.1* was obtained in the ABA-related module, confirming that L-theanine hydrolysis was the overall trend during withering and that this process was related to ABA.

To further understand the transcriptional regulatory mechanism of L-theanine hydrolysis, six candidate TFs (*CsWRKY40*, *CsbZIP*, *CsB3*, *CsbHLH*, *CsGRAS*, and *CsZF*) were identified from 344 genes related to *CsPDX2.1*. In tea plants, members of the *CsWRKY*, *CsbZIP*, *CsbHLH*, and *CsGRAS* families generally respond to abiotic stress (low temperature, dehydration, and salt stress) and exogenous hormone treatment, whereas members of the other two transcription factor families are less studied [25–28]. However, the transcriptional regulatory effects of these TFs on secondary metabolites that can affect the quality of tea have not previously been determined.

### **CsWRKY40** regulates the promoter of *CsPDX2.1*

Through a dual-luciferase assay, CsWRKY40 was identified based on its impressive regulatory effect (373.18-fold) on the *CsPDX2.1* promotor. EMSA was used to verify the physical interaction between CsWRKY40 and the *CsPDX2.1* promoter and to more precisely determine the location of the binding site in the promoter sequence. Most WRKY family proteins have been shown to bind to the cognate **cis-*acting* W box (T)(T)TGAC(C/T), and TGAC was the most conserved functional sequence [29, 30]. Some rare motifs bound by WRKY family proteins in other plants, such as the PRE4 element (TGCGCTT) and the sugar-responsive (SURE) element (TAAAGATTACTAATAGGAA), were not found in the *CsPDX2.1* promoter [31, 32]. EMSA results showed that CsWRKY40 bound *the *CsPDX2.1** promoter, and its binding sequence was TTGACC. In summary, *CsWRKY40* had a significant regulatory effect on the expression of *CsPDX2.1*. However, whether CsWRKY40 is the main or independent regulatory factor for L-theanine hydrolysis genes in tea plants requires further verification, perhaps through the use of transgenic technology.

*CsWRKY40* belongs to the WRKY group IIa family. In the plant kingdom, WRKY TFs are present in a variety of plants and have been demonstrated to participate in hormone signaling, biotic and abiotic stresses, and secondary metabolite metabolism [30, 33]. Wang et al. [28] reported that there were at least 56 *CsWRKY* genes in the tea plant genome. However, to date, only five *CsWRKYs* have been identified: *CsWRKY7* (group IId) [34], *CsWRKY26* (group I) [35], *CsWRKY2* (group I) [36], *CsWRKY31* (group IIb), and *CsWRKY48* (group IIc) [37]. Among them, *CsWRKY31* and *CsWRKY48* were found to inhibit the synthesis of EGCG3′′Me by binding to the promoter of genes related to EGCG3′′Me synthesis [37]. In the present work, we discovered and verified a member of the *CsWRKY* group IIa family for the first time, providing new insight into the regulatory effects of WRKY family members on secondary metabolites.

### Water loss is the triggering factor for L-theanine hydrolysis

**Figure 6:**
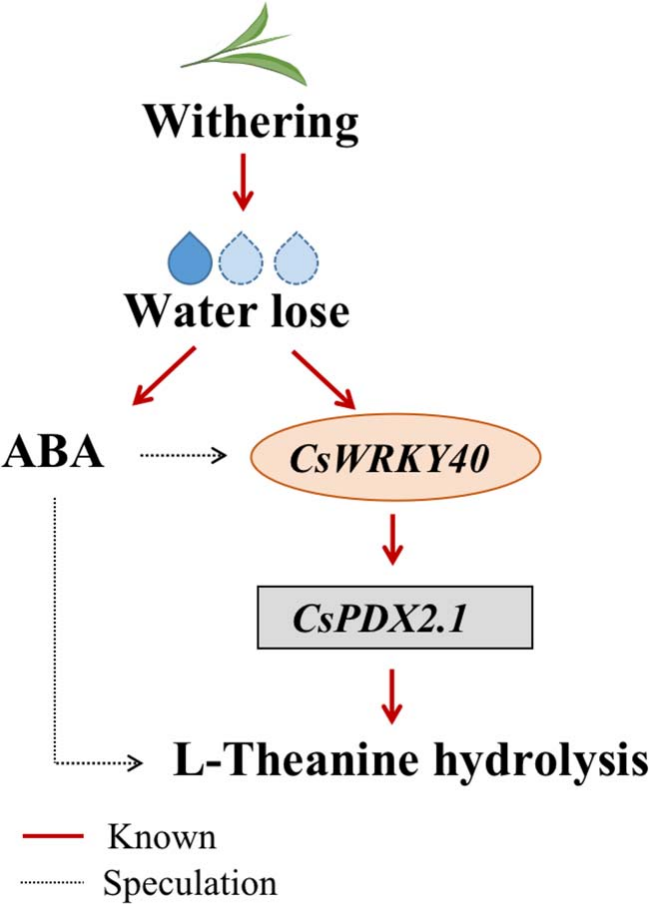
Upstream factors that regulate L-theanine hydrolysis in tea leaves during withering. The moisture content of detached tea leaves decreases with time. The expression of *CsWRKY40* increases, thus activating the promoter of *CsPDX2.1*, increasing the expression of the L-theanine hydrolase gene, and reducing L-theanine content. In addition, ABA accumulates and may inhibit L-theanine content by activating CsWRKY40 or other pathways. The solid red line represents the research content of this paper, whereas the dotted black line represents the speculated pathway.

During withering, the content of L-theanine in harvested tea is affected by a variety of environmental factors, such as light source [38] and temperature [39]. However, the most common and consistent observation is the loss of water in the withering process, which shrinks and softens the fresh tea leaves, making them suitable for subsequent processing. We therefore speculated that the loss of water may be the direct cause of L-theanine hydrolysis. Wang et al. [40] applied drought stress to two-year-old tea plants and found that the content of L-theanine decreased significantly with decreasing water content. In addition, throughout the tea manufacturing process, from high moisture content to complete dryness, L-theanine content gradually decreases [41]. To further determine the role of water in L-theanine hydrolysis, we established three water treatment models. Our results indicated that water loss plays a role as a trigger for L-theanine hydrolysis, and the expression levels of *CsPDX2.1* and *CsWRKY40* in the three treatments were consistent with this scenario (Fig. 5). In the withering treatment, the expression of *CsWRKY40* increased significantly after 12 h, which activated the expression of *CsPDX2.1* and caused a significant decrease in L-theanine content. Compared with the water retention treatment, the expression levels of *CsWRKY40* and *CsPDX2.1* gradually increased in the water loss treatment, resulting in the continuous hydrolysis of L-theanine. Interestingly, compared with the water retention treatment, ABA content continued to increase and remained at a higher level under the dehydration treatment. This is consistent with the results in previous reports [20, 42]. In the WGCNA analysis, *CsPDX2.1* was found in the module with the highest positive correlation to ABA, indicating that the expression pattern of *CsPDX2.1* during withering was significantly correlated with ABA content. More intuitively, the FPKM value of *CsPDX2.1* showed a trend similar to that of ABA content (Fig. S1). At the same time, in the dehydration treatment, the expression patterns of *CsWRKY40* and *CsPDX2.1* were consistent with the changes in ABA content. Previous work found that when exogenous ABA was applied to detached tea shoots, it significantly affected the content of L-theanine [43]. Given the vital role of ABA in plant response to abiotic stress, especially drought stress, we deduced that ABA may participate in L-theanine hydrolysis, and possible participation paths are shown in Fig. 6. In addition, *CsWRKY40* has high homology to *AtWRKY40*, **AtWRKY18*,* and *AtWRKY60* (Fig. S3), and previous studies have shown that *AtWRKY18* and *AtWRKY60* work cooperatively with *AtWRKY40* to regulate ABA-related drought responses [44]. This suggests that the response of the transcription factor *CsWRKY40* may be related to the water-induced ABA signal. Whether ABA is directly or indirectly involved in L-theanine hydrolysis through the upstream regulation of *CsWRKY40* remains to be determined.

## Materials and methods

### Plant materials and treatments

All tea materials (*C. sinensis* “Longjing 43”) were harvested from the Tea Research Institute at the Hangzhou Academy of Agricultural Sciences, China (N30°11′7.21″, E120°03′52.45″). Transcriptome data (GSE143971) were obtained from our previous work [20]. In brief, one bud and two leaves were withered under 25°C, 2000 lux, and 65% relative humidity for 24 h, and samples were collected at 0, 9, 15, and 24 h for transcriptome analysis.

In order to verify the dominant effect of water on L-theanine content and ABA content, a new batch of tender tea shoots with one bud and four leaves were picked on 29 April 2021. Tender stems without disease or wounding were cut with scissors and immediately inserted into culture bottles filled with water until treatment in the laboratory. All tea shoots were divided into three batches. The first batch of tea shoots was spread thinly onto white plates for withering. To establish the water retention treatment and the water loss treatment, the other two batches were inserted in water and 25% PEG6000 solution, respectively. The treated samples were incubated at 25°C and 65% humidity in the dark for 24 h in an artificial climate chamber. The second and third leaves of tea shoots were harvested at different time points (0, 3, 6, 9, 12, 18 and 24 h) for further experiments. The collected fresh samples were crushed with a grinder under liquid N_2_ and then stored at −80°C for later use.

### Weighted gene co-expression network analysis

The WGCNA R package was used for co-expression network analysis based on previous transcriptome data and physiological data [20]. A total of 26 524 genes (FPKM≥20) were classified into different modules. Combined with the changes in L-theanine and ABA contents, key genes were selected from the modules with the highest correlation coefficients. The soft power threshold was set to 14. Cytoscape 3.7.1 was used to describe the gene co-expression network.

### Tea genomic DNA and total RNA extraction

Genomic DNA and RNA were extracted from tea leaves using the CTAB (cetyltrimethyl ammonium bromide) method. For genomic DNA extraction, around 0.1 g fresh ground tea was added to 1 ml CTAB buffer (2% CTAB, 0.1 M Tris, 20 mM EDTA, 1.4 M NaCl, and pH 8.0) containing 2% β-mercaptoethanol (preheated to 65°C) and heated in a 65°C water bath for 10 min, then mixed well with 900 μl of chloroform/isoamyl alcohol (24:1, v:v). After centrifugation at 10000 rpm and 4°C for 10 min, the supernatant was transferred to a new tube, and an equal volume of chloroform and isoamyl alcohol mixture was added again. After centrifugation, 1/2 volume of 5 M NaCl and an equal volume of isopropanol were added to the supernatant, and the mixture was precipitated at −20°C for 1 h. The tube was then centrifuged at 12000 rpm and 4°C for 15 min. The precipitate was resuspended in 75% ethanol and centrifuged at 12000 rpm and 4°C for 2 min; the supernatant was removed, and the precipitate was washed again. After the ethanol had thoroughly evaporated, 100 μl TE buffer (10 mM Tris, 1 mM EDTA, and pH 8.0) was added to dissolve the pellet. Finally, 1 μl of solution was used to check the quality and concentration.

Total RNA was extracted by adding around 0.2 g leaves to 1 ml CTAB buffer (2% CTAB, 0.1 M Tris, 20 mM EDTA, 1.4 M NaCl and pH 8.0) containing 5% β-mercaptoethanol. After heating at 65°C for 15 min, 800 μl of chloroform/isoamyl alcohol (24:1, v:v) was added. After adequately mixing, the tube was centrifuged at 12000 rpm for 10 min. The supernatant was placed into a new 2-ml centrifuge tube, 800 μl of chloroform and isoamyl alcohol mixture was added, and the mixture was centrifuged at 12000 rpm for 10 min. The supernatant was transferred to a new 1.5-ml centrifuge tube, mixed with 1/5 volume of 12 M lithium chloride, and stored at 4°C for 12 h. After centrifugation at 10000 rpm and 4°C for 30 min, the precipitate was resuspended in 1 ml of 75% pre-cooled ethanol, centrifuged at 12000 rpm and 4°C for 5 min, and then washed again. When the ethanol had completely evaporated, the RNA pellet was dissolved in 20 μl sterile water.

### Dual-luciferase assay

A dual-luciferase assay was used to determine whether TFs had regulatory effects on the target promoter *in vivo*. Full-length genes and the promoter were cloned using Phanta Max Super-Fidelity DNA Polymerase (Vazyme) and then inserted into the pGreen II 0029 62-SK vector (SK) and the pGreen II 0800-LUC vector (LUC), respectively [45]. The primers for gene cloning and vector construction are listed in Supporting Information Table S1.

The methods of vector transformation and tobacco transfection were described by Wu et al. [46]. In brief, all recombinant SK and LUC vectors, as well as empty SK as a control, were electroporated into *Agrobacterium tumefaciens* GV3101. After the bacterial test, the successfully transformed bacteria were cultured on Luria-Bertani (LB) solid medium containing 50 μg ml^−1^ kanamycin and 25 μg ml^−1^ gentamycin. Bacterial samples were resuspended in infiltration buffer (10 mM MES, 10 mM MgCl_2_, 150 μM acetosyringone, and pH 5.6) when the maximum viability was reached, and the OD_600_ was then adjusted to 0.75. The infiltration solution, which was a mixture of TF and promoter suspension (v/v, 10:1), was carefully injected into the tobacco leaves (*Nicotiana benthamiana*). Three days after injection, intact leaf discs from infiltrated areas without injury were collected, thoroughly ground, and analyzed using dual-luciferase assay reagents (Vazyme).

### Phylogenetic analysis and sequence alignment

The amino acid sequences of CsWRKY40 and all *A. thaliana* Group II WRKY members were used for phylogenetic analysis. The amino acid sequence of CsWRKY40 was aligned with those of AtWRKY40 (AT1G80840), AtWRKY60 (AT2G25000), AtWRKY18 (AT4G31800), AcWRKY40 (PSS01081), and CcWRKY40 (CDO98227) to examine the domains of the Group IIa subfamily. MEGA X and DNAMAN software were used for phylogenetic tree construction and multiple sequence alignment, respectively.

### Electrophoretic mobility shift assay

The interaction of the CsWRKY40 protein and the *CsPDX2.1* promoter was assessed using an EMSA. The full-length **CsWRKY40* sequence* was cloned and inserted into the pGEX-4T-1 vector and then transformed into Rosetta-gami (DE3) pLys bacteria. The transformed strain was cultured at 37°C until the OD_600_ was 0.6–0.8 and then induced with IPTG (isopropyl β-D-1-thiogalactopyranoside, 0.3 mM) at 16°C for 20 h. The culture solution was centrifuged at 3000 rpm and 4°C for 15 min, and the precipitate was purified with a GST-tag Protein Purification Kit (Beyotime, Shanghai, China). The primers used are listed in Supporting Information Table S1.

EMSA was carried out according to the instructions of the LightShift Chemiluminescent EMSA kit (ThermoFisher Scientific). The 3′ biotin-labeled probes were synthesized by HuaGene (Shanghai, China), and double-stranded DNA probes were obtained by mixing and annealing the probes with their complementary chain. The probes without biotin label were used as cold probes to compete with labeled probes. The probes used in EMSA are listed in Supporting Information Table S2.

### Subcellular localization

The open reading frames (ORFs) of *CsWRKY40* and *CsPDX2.1* without stop codons were cloned and inserted into pCAMBIA2300-eGFP (enhanced green fluorescent protein). The primers for gene cloning are listed in Table S1. The constructed plasmids were transformed into *A. tumefaciens* GV3101 and then cultured on LB solid plates containing 50 μg ml^−1^ kanamycin. Activated bacterial samples were resuspended in infiltration buffer (10 mM MES, 10 mM MgCl_2_, 150 μM acetosyringone, and pH 5.6), and the OD_600_ was adjusted to ~0.75. *N. benthamiana* leaves were used for injection, infected leaf discs were collected after one day, and the eGFP signal was detected using a confocal laser scanning microscope (Nikon A1-SHS, Tokyo, Japan).

### cDNA synthesis and RT-qPCR analysis

A total of 1 g RNA per sample was used for cDNA synthesis. Genomic DNA was removed following the instructions of the PrimeScript 1st strand cDNA Synthesis kit (TaKaRa), and first-strand cDNA was synthesized for gene cloning and RT-qPCR.

RT-qPCR was performed using MonAmp ChemoHS qPCR Mix SYBR Green I (Monad) and a CFX96 instrument (Bio-Rad). The PCR reaction system prepared in the dark contained 10 μl MonAmp ChemoHS qPCR Mix, 2 μl diluted cDNA, 1 μl of each gene-specific primer (10 μM), and 6 μl RNAse-free water. After checking the melting curve, we aligned the PCR product sequence to ensure the specificity of the primers. *C. sinensis GAPDH* was used as an internal control for gene expression. Gene expression levels were analyzed by the 2^−ΔΔCt^ method. All gene-specific oligonucleotide primers were designed using Primer3 and are listed in Supporting Information Table S3.

### Moisture, L-theanine, and ABA measurements

The second and third leaves were used for physiological determination. Approximately 10 g of fresh tea samples were measured for moisture content using an infrared moisture meter (Sartorius MA35, Germany).

L-theanine extraction from fresh tea leaves was performed according to Cheng et al. [16]. L-theanine extraction solution (50 μl) was mixed with 200 μl *O*-phthalaldehyde (OPA) derivatization buffer (0.6 mM OPA acetonitrile solution [1 ml], 3-mercaptopropionic acid [125 μl], 0.4 M boric acid buffer [7 ml] and pH 10.2), and then 1 ml of 0.4 M boric acid buffer was added and diluted to 2 ml with distilled water. Afterwards, the derivative solution was filtered through a 0.45-μm filter and analyzed by HPLC (Shimadzu, Kyoto, Japan) with a Zorbax Eclipse AAA column (4.6×150 mm, 3.5 μm) at 40°C.

The mobile phase consisted of 40 mM dibasic sodium phosphate in water (pH 7.8) (A) and 45% methanol and 45% acetonitrile in water (B), and the flow rate was set to 1.5 ml min^−1^. Ten microliters of the filtrate was injected, and the gradient elution was performed as follows: the concentration of B increased from 5% (v/v) to 80% (v/v) over 18 min, increased to 100% (v/v) at 23 min, and was then maintained at 5% (v/v) for 7 min.

The endogenous ABA in tea leaves was extracted and detected according to the methods described by Xu et al. [20]. In brief, 0.1 g of crushed tea sample, 1 ml ethyl acetate, and 100 μl internal hormone standard (0.2 g L^−1^, D6-ABA) were mixed together, and the mixture was shaken overnight at 200 rpm and 4°C in the dark. After centrifugation at 12000 rpm and 4°C for 10 min, the supernatant was transferred to a fresh 10-ml centrifuge tube. The residue was extracted again using 1 ml of ethyl acetate with 2 h shaking and was centrifuged as described above. Finally, the supernatants were combined and dried under nitrogen. Then 0.5 ml 70% chromatographic methanol (v/v) was added to the supernatant and filtered through a 0.22-μm filter. The concentrations of endogenous hormones were analyzed with an LC–MS/MS instrument (Agilent 1260–6460) using an Agilent C18 column (150 mm × 2.1 mm, 3.5 μm). The mobile phase consisted of 0.1% formic acid (A) and methanol (B). The gradient elution was 40% B at 0 min, 100% B at 8 min, and 40% B at 10 min at a flow rate of 0.3 ml min^−1^. An electrospray ionization (ESI) source was used to detect mass spectra in negative ion multiple-reaction monitoring (MRM) mode.

## Acknowledgements

The authors are grateful to Prof. Xueren Yin of Zhejiang University for his precious advice on this project. This research was supported by the Natural Science Foundation of China (32072632).

## Author Contributions

P.X. and Y.W. designed the study. H.C., W.W., X.L., and Y.W. conducted the experiments and analyzed the data. H.C., Y.W., and P.X. wrote and edited the manuscript with input from all authors.

## Data availability

All the data generated in this study are included in this published article and its supplementary information.

## Conflict of interest statement

The authors declare no competing interests.

## Supplementary data

Supplementary data is available at *Horticulture Research* online.

## Supplementary Material

Web_Material_uhac025
