# Supplementary material for: Transcription factor CsWRKY40 regulates L-theanine hydrolysis by activating the CsPDX2.1 promoter in tea leaves during withering
Source: Hortic Res. 2022 Feb 19;9:uhac025. doi: 10.1093/hr/uhac025 (PMC9055099; doi:10.1093/hr/uhac025)
Supplement: Web_Material_uhac025 [file web_material_uhac025.docx]

**Supplementary materials**

**Transcription factor CsWRKY40 regulates L-theanine hydrolysis by activating the *CsPDX2.1* promoter in tea leaves during withering**

Haiyan Cheng ^1,2^, Wei Wu ^2,3^, Xiaofen Liu ^2,3^, Yuefei Wang ^1,2^, Ping Xu ^1,2,*^

^1^ *Institute of Tea Science, Zhejiang University, Hangzhou 310058, China*

^2^ *Key Laboratory of Horticultural Plant Growth, Development and Quality Improvement, Ministry of Agriculture, Hangzhou 310058, China*

^3^ *Zhejiang Provincial Key Laboratory of Horticultural Plant Integrative Biology, Zhejiang University, Hangzhou 310058, PR China*

***Correspondence Author:** [zdxp@zju.edu.cn](mailto:zdxp@zju.edu.cn)

**Table list**

Table S1. Primers for gene cloning and vector construction.

Table S2. 3’biotin probe, cold probe and mutant probe using for EMSA.

Table S3. Primers for qPCR quantification of *CsPDX2.1* and *CsWRKY40.*

**Figure list**

Fig. S1. Comparison of FPKM values of two L-theanine hydrolyzation-related genes in the blue module associated with ABA trait.

Fig. S2. Visualization of gene coexpression network.

Fig. S3. The phylogenetic tree analysis and sequence alignment of CsWRKY40.

**Table S1.** Primers for gene cloning and vector construction

| Gene | Vector | Primer | Sequence (5’>3’) |
| --- | --- | --- | --- |
| *CsPDX2.1*-promoter | 007VS | forward | CGCAGGAGTCCCATACCTTG |
|  |  | reverse | CTCCAGGGATGATGAGAGAGG |
| *CsPDX2.1*-promoter | pGreenII-Luc | forward | TCCTGCAGCCCGGGGGATCCCGCAGGAGTCCCATACCTTG |
|  |  | reverse | GTTTTTGGCGTCTTCCATGGTGTTGTTGCTTCAATTCAAC |
| *CsWRKY40* | pGreenII-SK | forward | GCTCTAGAACTAGTGGATCCATGGTAGATACAACACTCAG |
|  |  | reverse | ATAAGCTTGATATCGAATTCTCAGATATCATTTTCCCAAC |
| *CsbZIP* | pGreenII-SK | forward | GCTCTAGAACTAGTGGATCCATGGGAAATAGTGAAGAGAC |
|  |  | reverse | ATAAGCTTGATATCGAATTCTCAGCCAGCAGCCACAGCAT |
| *CsB3* | pGreenII-SK | forward | GCTCTAGAACTAGTGGATCCATGAGGAGTGAATCTGCGAA |
|  |  | reverse | ATAAGCTTGATATCGAATTCTTACCATGGAGCATCAACCT |
| *CsbHLH* | pGreenII-SK | forward | GCTCTAGAACTAGTGGATCCATGGCAGAGGAATTTCAAGC |
|  |  | reverse | ATAAGCTTGATATCGAATTCCTACCTGAATGTTCCTCCAA |
| *CsGRAS* | pGreenII-SK | forward | GCTCTAGAACTAGTGGATCCATGGAAACCTTAATCCGAGG |
|  |  | reverse | ATAAGCTTGATATCGAATTCTTAGTACTCTTTCACAGGCT |
| *CsZF* | pGreenII-SK | forward | GCTCTAGAACTAGTGGATCCATGAAGATCCAGTGTGATGT |
|  |  | reverse | ATAAGCTTGATATCGAATTCTCAACCTAGATCAGGAACAG |
| *CsWRKY40* | pGEX-4T-1 | forward | GGATCTGGTTCCGCGTGGATCCATGGTAGATACAACACTCAG |
|  |  | reverse | CGATGCGGCCGCTCGAGTCGACTCAGATATCATTTTCCCAAC |
| *CsPDX2.1* | eGFP | forward | ACGGGGGACGAGCTCATGGCCGTTGGTGTCCTC |
|  |  | reverse | GACTCTAGAGGATCCTTGAAATATAGGAAGATC |
| *CsWRKY40* | eGFP | forward | ACGGGGGACGAGCTCATGGTAGATACAACACTCAG |
|  |  | reverse | GACTCTAGAGGATCCGATATCATTTTCCCAACTTT |

**Table S2.** 3’biotin probe, cold probe and mutant probe using for EMSA

| probe | modified | Primer | Sequence (5’>3’) |
| --- | --- | --- | --- |
| 3’biotin probe | 3’biotin | forward | TTTATTTAAGAATTGACCGTAGATAACTTT |
|  | 3’biotin | reverse | AAAGTTATCTACGGTCAATTCTTAAATAAA |
| cold probe | - | forward | TTTATTTAAGAATTGACCGTAGATAACTTT |
|  | - | reverse | AAAGTTATCTACGGTCAATTCTTAAATAAA |
| mutant probe | 3’biotin | forward | TTTATTTAAGAAAAAAAAGTAGATAACTTT |
|  | 3’biotin | reverse | AAAGTTATCTACTTTTTTTTCTTAAATAAA |

**Table S3.** Primers for qPCR quantification of *CsPDX2.1* and *CsWRKY40*

| Gene | Primer | Sequence (5’>3’) |
| --- | --- | --- |
| *CsGAPDH* | forward | TTGGCATCGTTGAGGGTCT |
|  | reverse | CAGTGGGAACACGGAAAGC |
| *CsPDX2.1* | forward | TGCTAGGGACTGCCTTTCAT |
|  | reverse | TTCTCCAACTGCAACAATGC |
| *CsWRKY40* | forward | TGCCAACAAGTCCAATATTCTG |
|  | reverse | TCCCAACTTTCTATCGGATCAT |


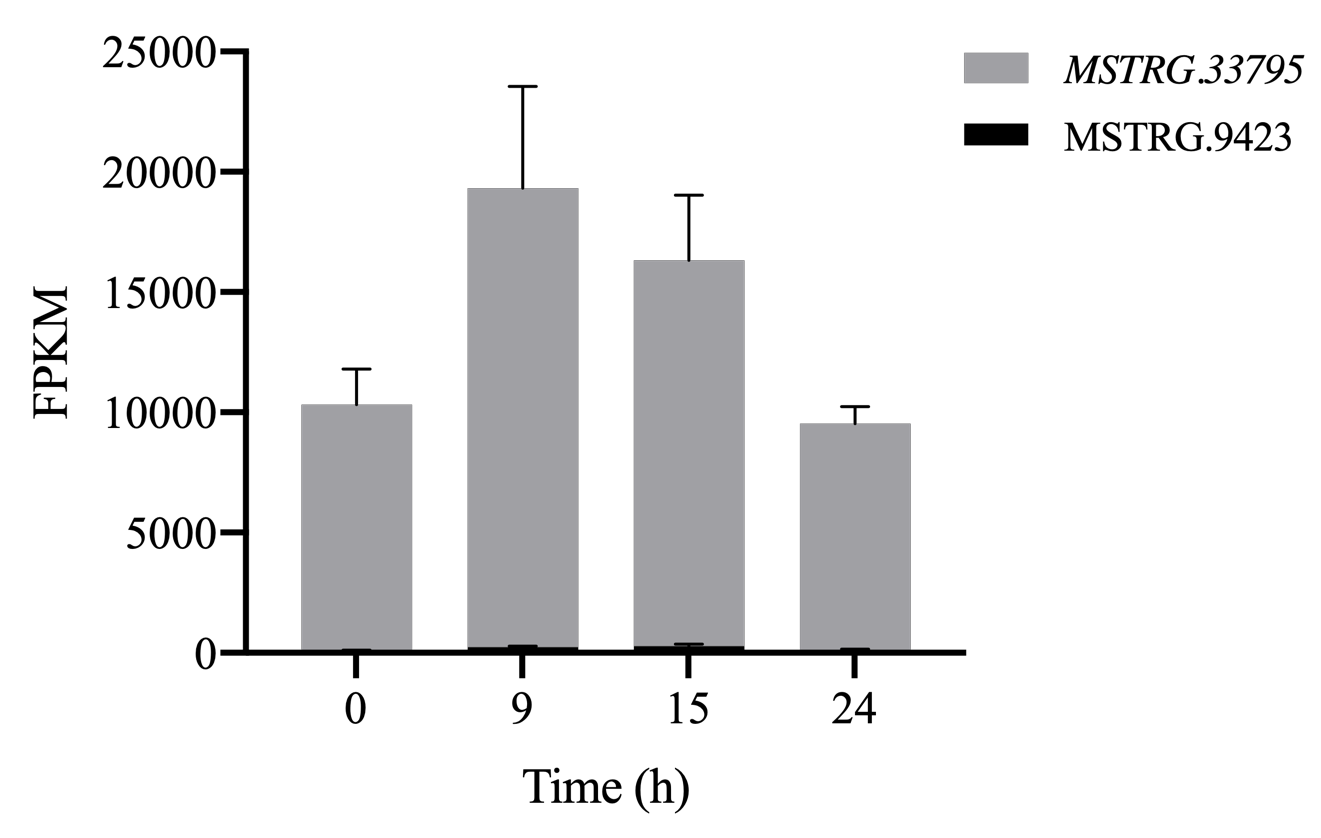


**Fig. S1.** Comparison of FPKM values of two L-theanine hydrolyzation-related genes in the blue module associated with ABA trait. The horizontal axis represents the time for tea withering.


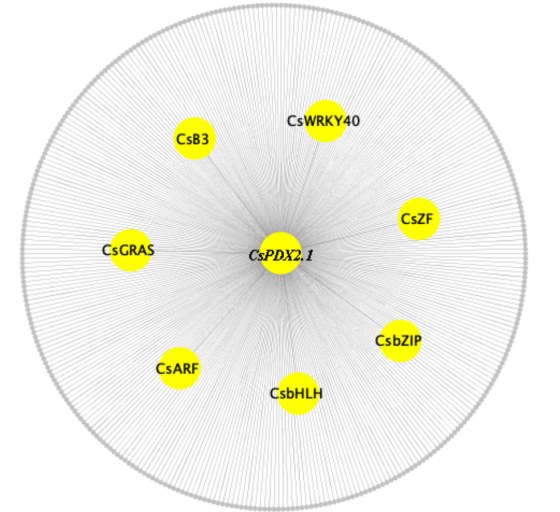


**Fig. S2.** Visualization of gene coexpression network. 7 candidate transcription factors were screened out of 344 genes related to *CsPDX2.1*. Cytoscape was used to construct network.


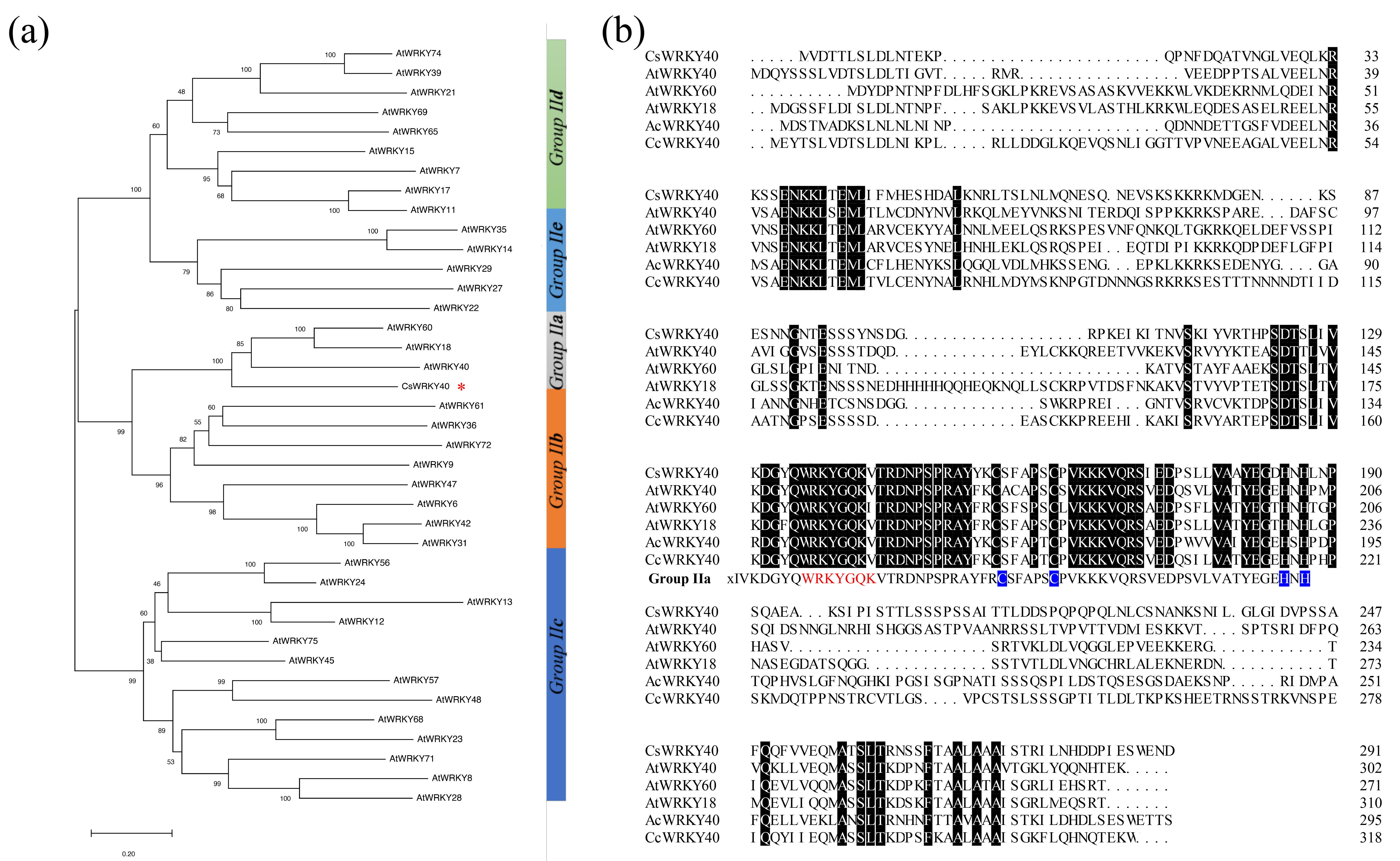


**Fig. S3.** The phylogenetic tree analysis and sequence alignment of CsWRKY40. (a) Comparison with *Arabidopsis* group II WRKY proteins indicates that CsWRKY40 was a group IIa protein, and the highest homology with AtWRKY40. A phylogenetic tree was constructed using MEGAX. (b) Sequence alignment of CsWRKY40 protein with homologous WRKYs from other plants. The WRKY domain consensus for group IIa are shown, red highlight and blue shadow represent the WRKY domain and the zinc finger motif, respectively. DNAMAN was used for sequence alignment.
